# Therapeutic effect of a histone demethylase inhibitor in Parkinson’s disease

**DOI:** 10.1038/s41419-020-03105-5

**Published:** 2020-10-28

**Authors:** Ming-Dao Mu, Zhong-Ming Qian, Sheng-Xi Yang, Kang-Lin Rong, Wing-Ho Yung, Ya Ke

**Affiliations:** 1 School of Biomedical Sciences, Faculty of Medicine, The Chinese University of Hong Kong, Shatin, NT, Hong Kong SAR, China; 2 Gerald Choa Neuroscience Centre, The Chinese University of Hong Kong, Shatin, NT, Hong Kong SAR, China; 3 Institute of Translational and Precision Medicine, Nantong University, Nantong 226001, China

**Keywords:** Cell death in the nervous system, Parkinson's disease

## Abstract

Iron accumulation in the substantia nigra is recognized as a hallmark of Parkinson’s disease (PD). Therefore, reducing accumulated iron and associated oxidative stress is considered a promising therapeutic strategy for PD. However, current iron chelators have poor membrane permeability and lack cell-type specificity. Here we identified GSK-J4, a histone demethylase inhibitor with the ability to cross blood brain barrier, as a potent iron suppressor. Only a trace amount of GSK-J4 significantly and selectively reduced intracellular labile iron in dopaminergic neurons, and suppressed H_2_O_2_ and 6-OHDA-induced cell death in vitro. The iron-suppressive effect was mainly mediated by inducing an increase in the expression of the iron exporter ferroportin-1. In parallel, GSK-J4 rescued dopaminergic neuron loss and motor defects in 6-OHDA-induced PD rats, which was accompanied by reduction of oxidative stress. Importantly, GSK-J4 rescued the abnormal changes of histone methylation, H3K4me3 and H3K27me3 during 6-OHDA treatment although the iron-suppressive and neuroprotective effects were sensitive to H3K4me3 inhibition only. Also, upregulating H3K4me3 increased ferroportin-1 expression and neuroprotection. Taken together, we demonstrate a previously unappreciated action of GSK-J4 on cell-specific iron suppression and neuroprotection via epigenetic mechanism. Compared with conventional iron chelators, this compound has a stronger therapeutic potential for PD.

## Introduction

Parkinson’s disease (PD) is the second most common neurodegenerative disease, which affects millions of people worldwide and has been posing a heavy burden on the healthcare system in many regions^1,2^. PD patients typically suffer from resting tremor, rigidity, bradykinesia, and posture instability. It is widely believed that most typical PD symptoms are the results of dopaminergic (DA) neuron loss in the midbrain substantia nigra (SN). At present, PD still remains incurable and only symptomatic treatments, such as L-DOPA medication and deep brain stimulation, are available. With disease progression, however, these symptomatic medications lose efficacy^2,3^. There is thus a significant unmet need for new medications capable of slowing or preventing PD progression by blocking SN neuron death.

Intense research effort in the past has led to the discovery of several central hallmarks in PD, including, alpha-synuclein aggregation, mitochondrial dysfunction, and iron accumulation^2,4,5^. Among all, iron accumulation in SN is one of the most invariable pathological findings in PD^4,6–8^. Iron is vital to the function of a wide variety of proteins. However, labile iron, through Fenton reaction, can generate free radicals from by-products of mitochondrial respiration, triggering oxidative stress^9^. Although it is known that iron accumulation can induce oxidative stress and therefore is toxic, the exact role of iron accumulation in SN of PD patients still remains largely unknown. According to transcranial sonography and other live brain iron detection methods, it is believed that increased level of iron in SN can be one of the earliest pathological changes in PD^6,7,10^. Moreover, increased non-transferrin-bound iron (NTBI) uptake via divalent metal transporter 1 (DMT1)^11^, transferrin-bound iron (TBI) uptake into mitochondria mediated by transferrin/transferrin receptor 2 (Tf/TfR2)^12^ and reduced iron export via ferroportin-1 (Fpn1), the sole iron export protein identified^13^, have been found associated with iron accumulation in PD^7,10^. The discovery of the genetic association between iron-related proteins and PD occurrence further supports the hypothesis that iron dysregulation could play a key role in causing PD.

A number of reports have demonstrated that oxidative damages, potentially caused by iron-induced oxidative stress, may appear to be one of the earliest pathological changes in PD^6,8^. Recent findings that iron accumulation and oxidative stress may trigger and accelerate pathological changes of PD, together with the discovery of the association of a number of iron-related and oxidative stress-related genes with PD, provide further supporting evidence that iron accumulation and elevated oxidative stress may be early PD pathogenic events causing DA neurons loss^2,8,14^. Consistently, treatments with iron chelators and antioxidants have been found effective in PD, suggesting that iron chelators and antioxidants might be promising targets for disease-modifying treatment of PD^2,15,16^. However, iron-suppressing treatment has yet been widely adopted due to major limitations concerning blood–brain-barrier (BBB) permeability and cell-type specificity of current iron-suppressing drugs^17,18^.

GSK-J4 is a newly developed cell-permeable prodrug that, once entered into cells, can be hydrolyzed by esterase to GSK-J1, a potent inhibitor of H3K27me3/me2 and H3K4me3/me2 histone demethylase (also known as KDM6A/B and KDM5B/C, respectively)^19,20^. A number of reports have suggested a possible application of GSK-J4 in cancer treatment and inflammation suppression based on its inhibitory effects on histone demethylases^21^. In some studies, silencing histone demethylases, such as KDM5C, the most abundant form of KDM5 in the brain, is associated with beneficial effect in neurodegenerative diseases such as Huntington’s disease^22^. Due to the capability of removing Fe^2+^ from targeted demethylases, GSK-J4/1 has also been suggested to be a possible iron chelator^19^. Therefore, in this study, based on the well-established models of PD, experiments were designed to investigate if GSK-J4 treatment is beneficial in models of PD and to elucidate the detailed underlying mechanisms.

## Results

### Selective iron suppression effect of GSK-J4 on DA neurons

**Fig. 1:**
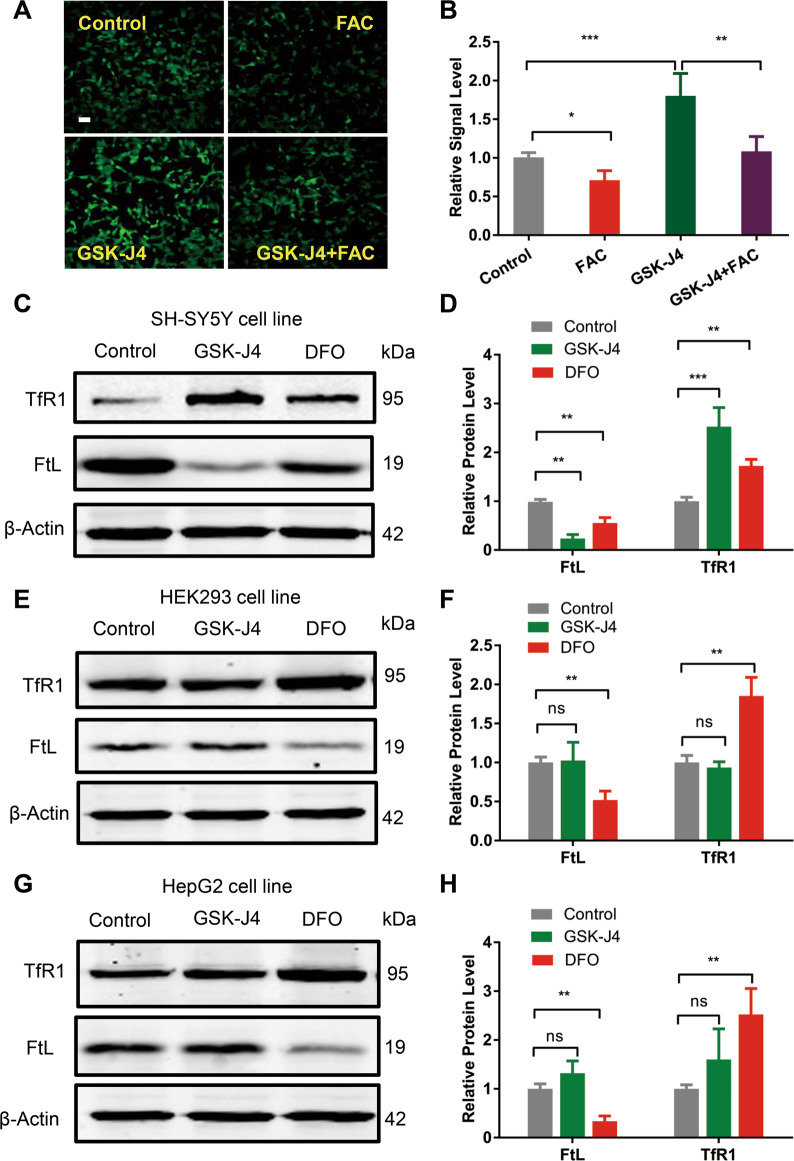
GSK-J4 displays a cell-specific iron suppressive capability. **a**, **b** SH-SY5Y cells treated with the iron supplement FAC (50 μg/ml) for 12 h displayed a lower calcein fluorescence level than the control group. However, calcein fluorescence increased with the addition of 0.5 μM GSK-J4, and the addition of FAC (50 μg/ml) inhibited the GSK-J4-mediated increase of calcein fluorescence. Scale bar, 50 μm. **c**, **d** GSK-J4 treatment (0.5 μM, for 24 h) reduced the iron storage protein ferritin (FtL) level in SH-SY5Y cell line as revealed by western blotting but did not cause a significant change in the expression of FtL in the HEK293 cell line **e, f** and in the HepG2 cell line **g**, **h**. In addition, the common iron chelator deferoxamine (DFO, 50 μM, for 24 h) caused a decrease of FtL in all tested cell lines. The relative protein levels of FtL was normalized to β-Actin. *P < 0.05; **P < 0.01; ***P < 0.001; ns means no significant difference. Data are presented as mean ± SEM. *n* = 3–4 for each group.

To examine the effects of GSK-J4 on the potentially toxic intracellular labile iron pool (LIP) in cells, a calcein-AM assay was performed. Since the fluorescence of calcein is partially quenched upon binding to iron, compounds that displace intracellular iron from its complex with calcein would result in an increased fluorescence signal. As shown in Fig. 1a, b, SH-SY5Y cells treated with the iron supplement FAC (50 μg/ml) for 12 h displayed a lower calcein fluorescence level than the control group (*P* < 0.05). However, calcein fluorescence increased with the addition of 0.5 μM GSK-J4 (*P* < 0.001, compared with control group), suggesting a decrease in free labile iron in the SH-SY5Y cells with the treatment of GSK-J4. Moreover, the addition of FAC (50 μg/ml) inhibited the GSK-J4-mediated increase of calcein fluorescence (*P* < 0.01, compared with GSK-J4 group), further indicating that the elevated calcein signal by GSK-J4 was caused by the iron suppressing effect of GSK-J4.

We further characterized the effect of GSK-J4 on cellular iron homeostasis by examining the expressions of the iron importer protein TfR1 and the iron storage protein ferritin light chain (FtL). As shown in Fig. 1c, d in SH-SY5Y cells treated with the iron chelator deferoxamine (DFO, 50 μM), TfR1 synthesis was increased compared to untreated control cells (*P* < 0.01), whereas synthesis of FtL is strongly inhibited (*P* < 0.01). Interestingly, similar to DFO, only a trace amount of GSK-J4 (0.5 μM) treatment caused a significant increase of TfR1 (*P* < 0.001) and a decrease of FtL (*P* < 0.01). These findings are consistent with that under the condition of free labile iron depletion, IRP1 stabilizes TfR1 mRNA to prolong its half-life, while inhibiting the translation of FtL mRNA^23,24^. These effects enhance iron uptake into cells and stops it from being sequestered away for storage. However, the effects of GSK-J4 treatment on FtL and TfR1 were not found in HEK293 (Fig. 1e, f) and HepG2 cell line (Fig. 1g, h). In contrast, the classical iron chelator DFO (50 μM) caused a significant increase of TfR1 (*P* < 0.01, compared with each control group) and a significant decrease of FtL (*P* < 0.01, compared with each control group), in all these cell lines (Fig. 1c–h). In addition, the iron suppression effect was also found in the MES23.5 cell line (Supplementary Fig. 1a, b). Since SH-SY5Y and MES23.5 both are widely used DA neuronal cell lines, these results indicate that GSK-J4 has a selective iron suppressing effect on DA neurons.

### GSK-J4 displays a potent neuroprotective effect in 6-OHDA-induced PD model in vitro

**Fig. 2:**
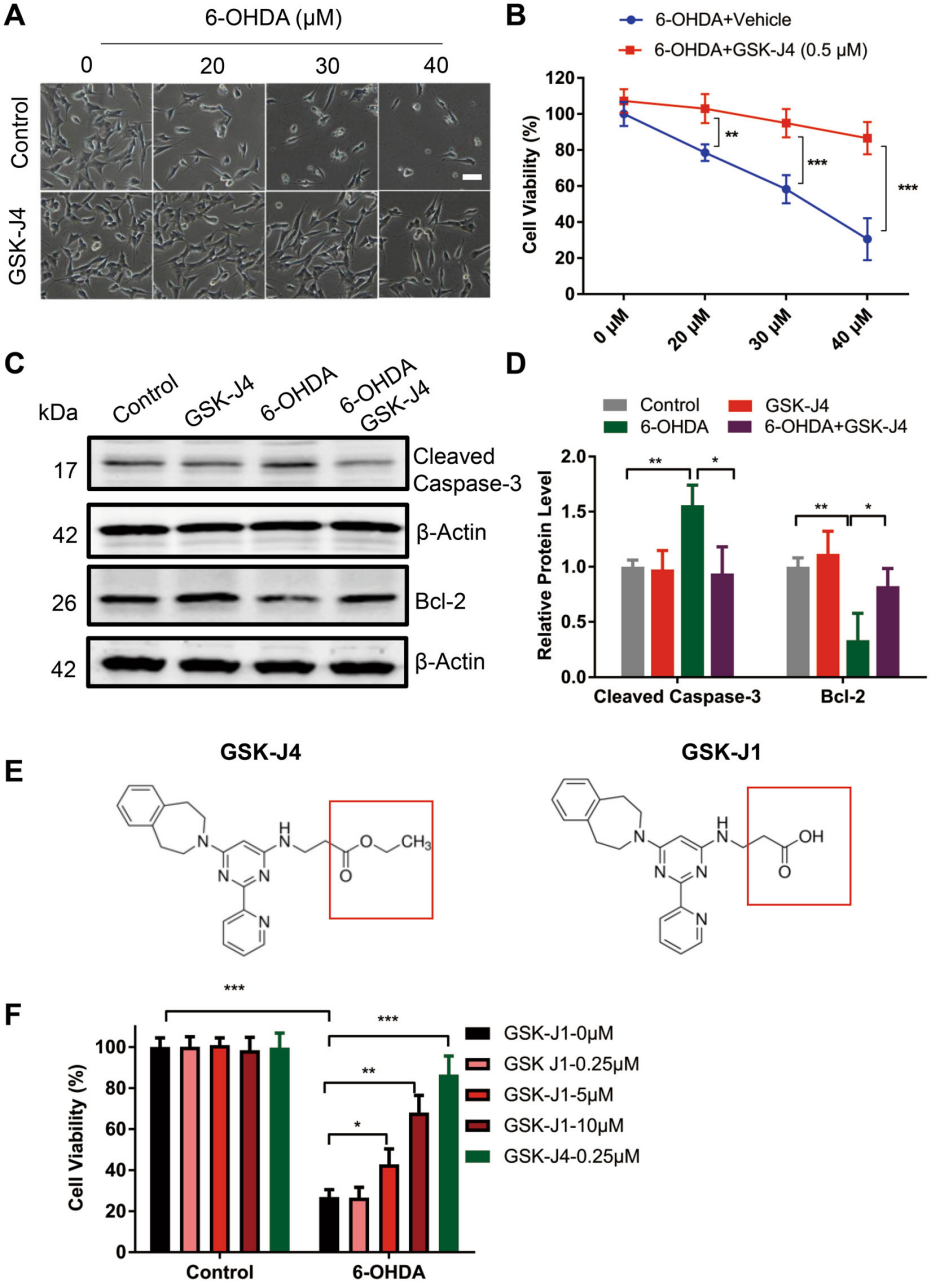
GSK-J4 exerts a powerful neuroprotective effect in in vitro PD model. **a** The cell morphology of undifferentiated SH-SY5Y under 6-OHDA-treatment with or without GSK-J4 (0.5 μM, for 24 h). Scale bar, 50 μm. **b** Pretreatment with GSK-J4 (0.5 μM, 1 h before 6-OHDA treatment) significantly inhibited the decrease in cell viability under 6-OHDA treatment. **P < 0.01; ***P < 0.001. Data are presented as mean ± SEM. *n* = 4–6 for each group. **c** and **d** Consistently, GSK-J4 pretreatment (0.5 μM, 1 h before 6-OHDA treatment) inhibited the increase in cleaved caspased-3 and prevented the decrease in Bcl-2 under 6-OHDA-treatment (25 μM, 24 h), while GSK-J4 treatment alone caused no significant change in level of cleaved caspased-3 and Bcl-2. *P < 0.05; **P < 0.01. Data are presented as mean ± SEM. *n* = 3–4 for each group. **e** Chemical structure of GSK-J1 and GSK-J4. **f** In SH-SY5Y cells, pretreatment with GSK-J1 (0–10 μM, 1 h) or GSK-J4 (0.25 μM, 1 h) inhibited 6-OHDA-induced cell death. *P < 0.05; **P < 0.01; ***P < 0.001. Data are presented as mean ± SEM. *n* = 5 for each group.

Considering the cell-type specificity of the iron-suppressing effect of GSK-J4, we hypothesize that it is beneficial in PD. To investigate the effect of GSK-J4 on a PD model in vitro, SH-SY5Y cells were treated with the neurotoxin 6-hydroxydopamine (6-OHDA). As shown in Fig. 2a, 6-OHDA induced a dose-dependent shrinkage, detachment, and cell death in SH-SY5Y cells in 24 h. Compared with 6-OHDA-treated cells, pre-treatment with GSK-J4 (0.5 μM) clearly protected cells against 6-OHDA toxicity, even at a high concentration of 6-OHDA (40 μM). The protective effect of GSK-J4 was also quantitatively assessed by the MTT assay (Fig. 2b). For example, upon 40 μM of 6-OHDA treatment for 24 h, 0.5 μM of GSK-J4 significantly increased cell viability from 30.5 ± 10.9 to 86.6 ± 8.4% (*P* < 0.001). On the other hand, treatment with GSK-J4 alone (0.5 μM) did not have a significant effect on morphology and cell viability. These data suggest that GSK-J4 showed a powerful neuroprotection in the 6-OHDA-treated SH-SY5Y cells. We also examined the expressions of two important markers of cell apoptosis, namely cleaved Caspase-3 and Bcl-2 by Western blot. 6-OHDA treatment increased the level of cleaved caspase-3 to 160% (Fig. 2c, d) and decreased the level of Bcl-2 to 51% when compared with the control group, while pretreatment with GSK-J4 significantly prevented these changes (Fig. 2c, d). These data suggest that GSK-J4 decreased the 6-OHDA-induced apoptosis in SH-SY5Y cells.

GSK-J4 is a highly cell permeable ethyl ester derivative of GSK-J1^19^ (Fig. 2e). To compare the neuroprotective effects of these two compounds, SH-SY5Y cells were pretreated with GSK-J1 (0–10 μM) 1 h before the 6-OHDA (30 μM, 12 h) treatment, and MTT assay was performed. Consistently, GSK-J1 prevented 6-OHDA-induced cell death (*P* < 0.01, comparing the 6-OHDA group with 6-OHDA+GSK-J1 group) (Fig. 2f), but a concentration of 10 μM GSK-J1, that is, almost 40 times that of GSK-J4 at 0.25 μM, was required in order to display the same neuroprotective effect. The difference is therefore attributable to the high cell membrane permeability of GSK-J4 compared with GSK-J1.

### Neuroprotective effect of GSK-J4 in PD models in vivo

**Fig. 3:**
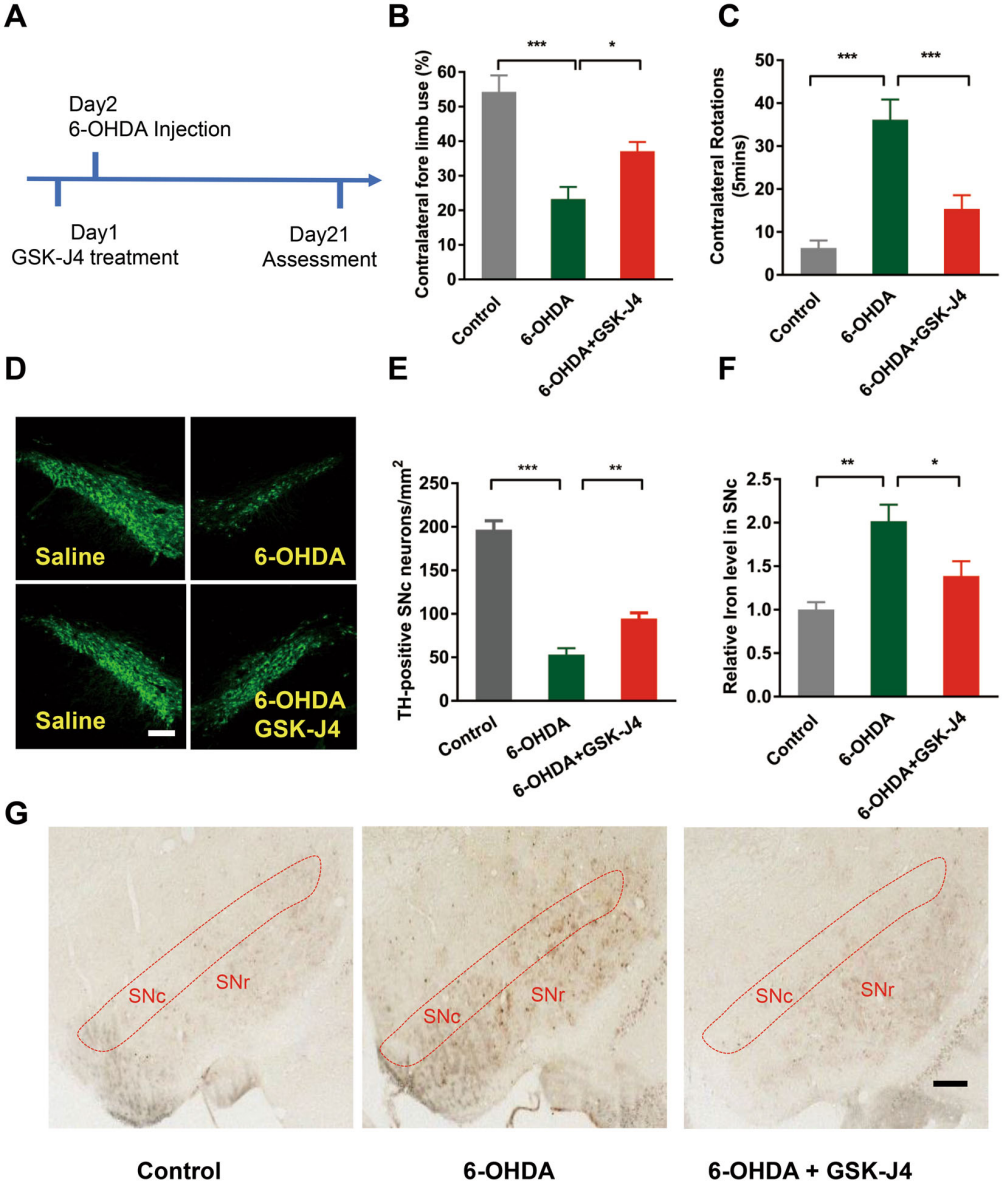
GSK-J4 exhibits neuroprotection in 6-OHDA-induced PD animal model. **a** Experimental scheme of unilateral 6-OHDA injection-induced PD model. **b** The cylinder test showed that GSK-J4 rescued the unilateral 6-OHDA-induced asymmetry of the forelimbs. **c** Consistent with the above, the apomorphine-induced rotation in the unilateral 6-OHDA-treated rat was suppressed by the treatment of GSK-J4. **d**, **e** Tyrosine hydroxylase staining showed that MFB injection of 6-OHDA caused a significant decrease of the TH-positive neuron in SN (−4.80 to −6.12 mm from Bregma), while GSK-J4 treatment rescue the level of TH neuron. Scale bar, 100 μm. **f**, **g** 6-OHDA treatment caused a significant increase in iron accumulation in the SN, which was prevented by the treatment of GSK-J4, as reflected by Perls’-DAB staining. *P < 0.05; **P < 0.01; ***P < 0.001. Data are presented as mean ± SEM. Scale bar, 200 μm. *n* = 8 for the behavioral test, and *n* = 6 for TH staining.

To explore the neuroprotective and therapeutic effects of GSK-J4 in in vivo PD model, SD rats were mico-injected with 6-OHDA (8 μg/2 μl) at the medial forebrain bundle (MFB) in the right side of the brain. GSK-J4 (1 μg/1 μl) was injected into MFB one day before the 6-OHDA administration (Fig. 3a). Three weeks after injection, the relevant behavior tests were performed before sacrifice of the animals for histological and biochemical assessments. In the cylinder test, unilateral 6-OHDA injection decreased the use of the contralateral forelimb to about 20% while GSK-J4 treatment partially but significantly rescued this motor defect, in which the use of contralateral forelimb was almost 40% (Fig. 3b). In the apomorphine-induced rotation test, GSK-J4-treated rat exhibited far fewer number of contralateral rotation (15 rpm) in 5 min when compared with the unilateral 6-OHDA-treated rat, which was about 36 rpm in the same period of time (Fig. 3c). These results indicate that GSK-J4 can prevent the 6-OHDA-induced motor defects in rats. At the same time, based on tyrosine hydroxylase (TH) staining, it was found that 6-OHDA injection caused a significant decrease in the number of TH-positive neurons in SN, which was about 25% of the saline-treated side. With GSK-J4 treatment, 50% of TH-positive neurons survived after 6-OHDA injection indicating a neuroprotective effect of GSK-J4 (Fig. 3d, e).

To establish the link between the beneficial effect of GSK-J4 and iron level in the PD animal model, we first asked whether iron was altered in the unilateral 6-OHDA-induced rat model. Iron staining (Fig. 3f, g) showed that the number of iron-positive cells significantly increased in the 6-OHDA-lesioned SN, indicating increased iron content after 6-OHDA treatment. However, the treatment of GSK-J4 significantly inhibited this increase of iron, suggesting that GSK-J4 can reduce iron accumulation in response to 6-OHDA treatment.

### Anti-oxidative stress is involved in the neuroprotection of GSK-J4

Iron-induced oxidative stress as a result of iron accumulation is regarded as an important cause of neurodegeneration in many experimental PD models. Given the iron suppression effect of GSK-J4 that we observed, we investigated whether anti-oxidative stress plays a role in the neuroprotective effect of GSK-J4 in PD model.

**Fig. 4:**
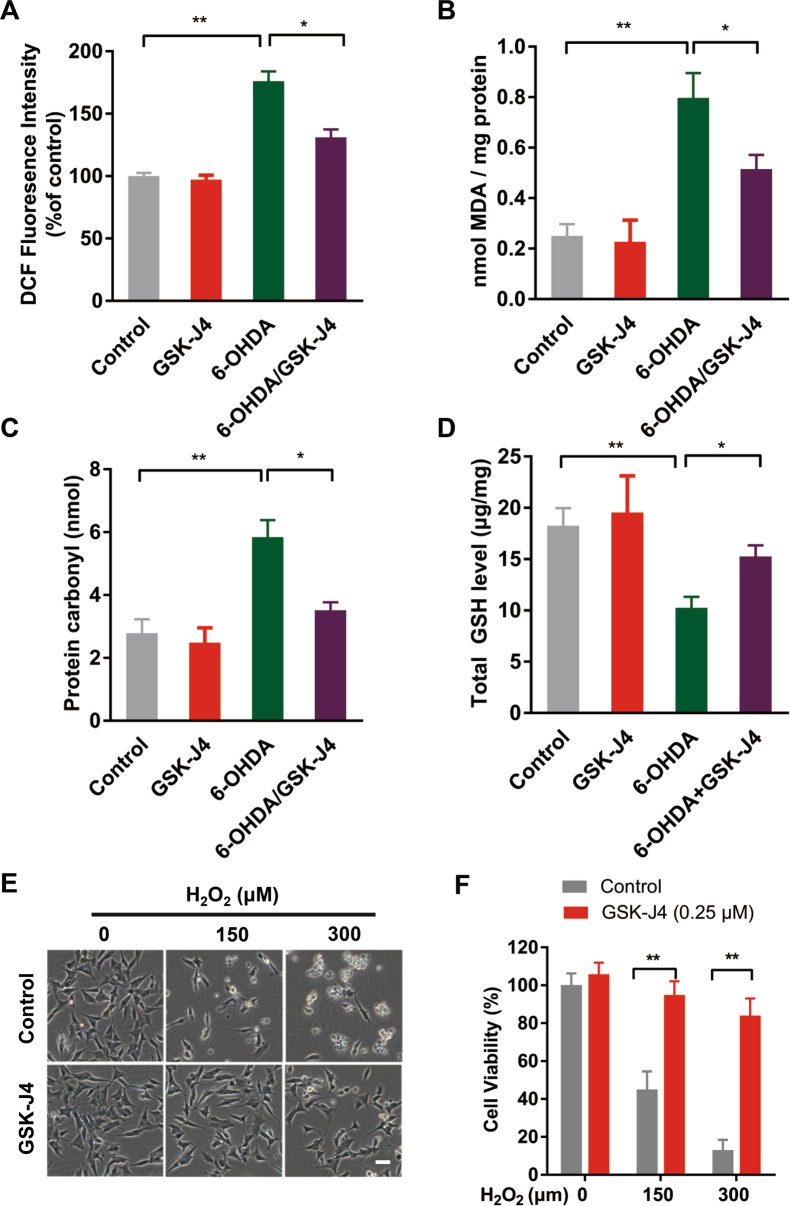
Antioxidant effects of GSK-J4 in the 6-OHDA-induced PD model. MFB injection of GSK-J4 prevented 6-OHDA-induced increase of **a** ROS, **b** MDA, and **c** Protein carbonyl in the SN. Besides, **d** the reduction of GSH in SN caused by 6-OHDA was rescued by GSK-J4 treatment. In addition, GSK-J4 treatment alone had no significant effect on these oxidative stress markers. *P < 0.05; **P < 0.01. Data are presented as mean ± SEM. *n* = 4 for each group. In addition, in SH-SY5Y cells, pretreatment with GSK-J4 (0.25 μM, 1 h) prevented **e** H_2_O_2_-induced cell death, Scale bar, 50 μm **f** as reflected by MTT assay. **P < 0.01. *n* = 5 for each group.

We determined the levels of different oxidative stress markers in the SN of 6-OHDA-treated rats in the absence and presence of GSK-J4 pre-administration. It was found that GSK-J4 could significantly suppress the 6-OHDA-induced increase of reactive oxygen species (ROS) production, as measured by DCF fluorescence, from 176 ± 7.9% to 131 ± 6.3% (Fig. 4a), the increase of MDA level from 0.79 ± 0.10 to 0.52 ± 0.12 nmol (Fig. 4b), the increase of protein carbonyl level from 5.84 ± 0.54 to 3.52 ± 0.25 nmol (Fig. 4c) and the decrease of total GSH level from 10.27 ± 1.05 to 15.27 ± 1.08 μg/mg (Fig. 4d). These results indicate that GSK-J4 acts as a strong antioxidant in the 6-OHDA-induced PD model.

To further confirm the antioxidant nature of GSK-J4, we tested the effect of this compound on SH-SY5Y cells treated with H_2_O_2_. We found that GSK-J4 at 0.25 μM prevented H_2_O_2_-induced changes in cell death reflected by changes in morphology (Fig. 4e). In both 150 and 300 μM concentrations of H_2_O_2_, pretreatment with GSK-J4 maintained a high level of cell viability, confirmed with MTT assay (Fig. 4f).

### GSK-J4 prevents abnormal histone methylation in 6-OHDA model

**Fig. 5:**
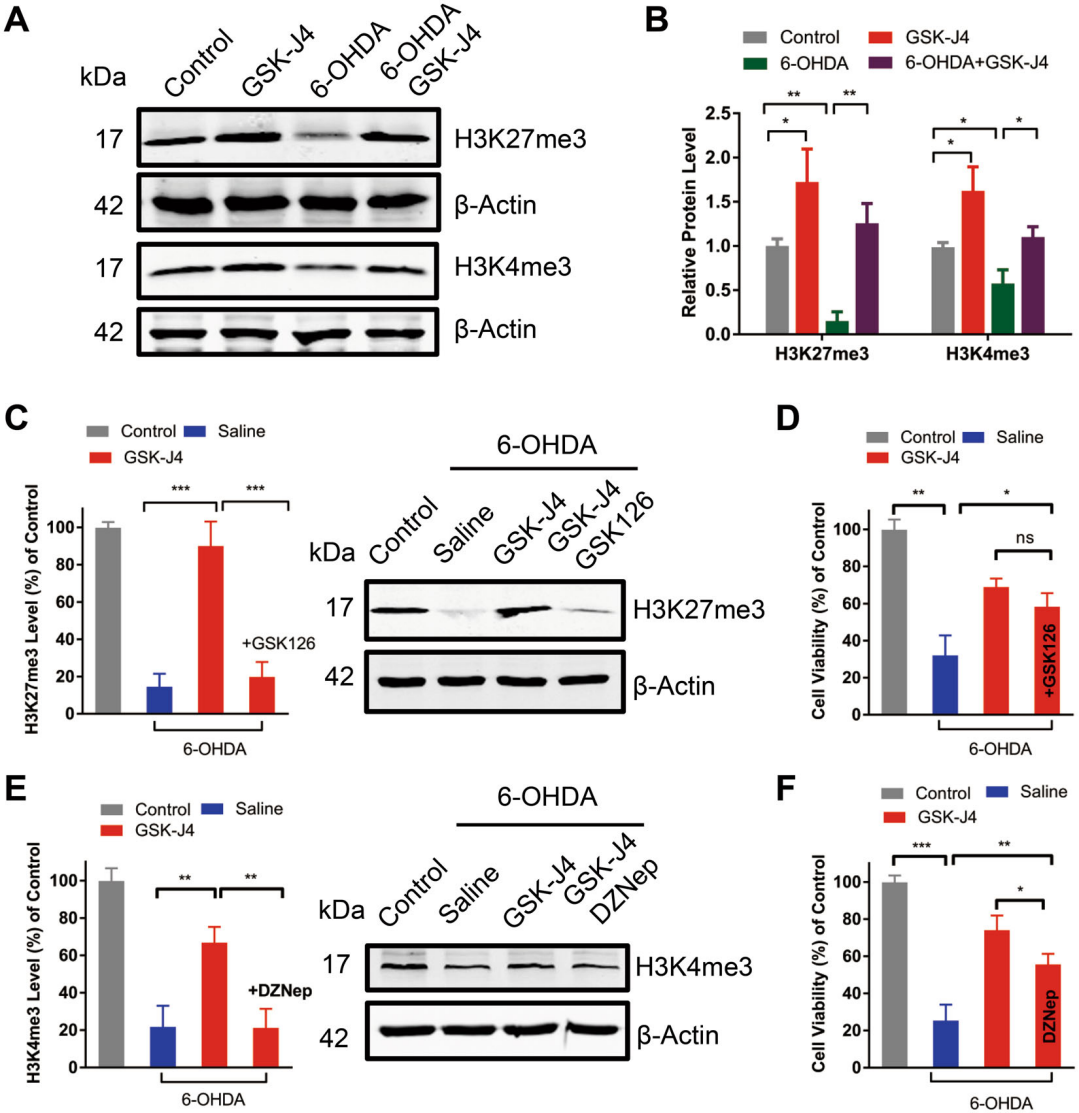
GSK-J4 rescues 6-OHDA-induced abnormal histone methylation. **a**, **b** In SH-SY5Y cell line, GSK-J4 treatment (0.5 μM, for 24 h) increased not only the basal levels of H3K27me3 and H3K4me3 but also rectified the decrease in their levels under 6-OHDA-treatment (25 μM, for 24 h). **c**, **d** SH-SY5Y cells were pretreated with GSK-J4 (0.5 μM, for 1 h), followed by the treatment of 6-OHDA (25 μM, for 24 h) with or without GSK126 (1 μM, a specific inhibitor of H3K27me3). As reflected by Western blot **c**, H3K27me3 was inhibited under the treatment of GSK126. ***P < 0.001. Data are presented as mean ± SEM. *n* = 3 for each group. However, **d** MTT assay showed that no inhibition effect from GSK126 was observed on the neuroprotection of GSK-J4 in the 6-OHDA-induced model. **e**, **f** SH-SY5Y cells were pretreated with GSK-J4 (0.5 μM, 1 h), followed by the treatment of 6-OHDA (25 μM, 24 h) with or without DZNep (1 μM, decreasing H3K4me3 level). H3K4me3 was inhibited under the treatment of DZNep **e**, while MTT assay revealed a partial inhibitory effect by DZNep **f**. *P < 0.05; **P < 0.01; ***P < 0.001. Data are presented as mean ± SEM. ns means no significant difference. *n* = 3–4 for each group.

As a selective inhibitor of histone demethylases including KDM5 and KDM6, GSK-J4 could increase the levels of H3K4me3 and H3K27me3, respectively^19,20^. Therefore, apart from the effect on iron level and oxidative stress, we asked whether the effects of GSK-J4 on H3K27me3 and H3K4me3 contribute to its therapeutic action in PD animals. As shown in Fig. 5a, b, SH-SY5Y cells treated with GSK-J4 (0.5 μM) for 24 h exhibited an increase in the level of H3K27me3 (1.72-fold of control group), and an increase in the level of H3K4me3 (1.62-fold of control group) in SH-SY5Y cells. Moreover, there were significant reductions in H3K27me3 (*P* < 0.01) and H3K4me3 (*P* < 0.05) under the 6-OHDA (25 μM) treatment when compared with the control group but pretreatment with GSK-J4 significantly attentuated this decrease (*P* < 0.05, when compared with the 6-OHDA-lesioned group, Fig. 5a, b). These data reveal abnormal levels of H3K4me3 and H3K27me3 in PD model that could be rectified by GSK-J4.

### H3K4me3 contributes to the neuroprotection of GSK-J4 treatment on the 6-OHDA-induced PD model

To further explore and distinguish the roles of H3K27me3 and H3K4me3 in the protective effect of GSK-J4 on the 6-OHDA-induced PD model, two sets of experiments were performed. First, SH-SY5Y cells were treated with GSK-J4 (0.5 μM) followed by treatment with a highly selective EZH2 methyltransferase inhibitor (GSK126, 1 μM) that could potently inhibit H3K27me3^25,26^. As shown in Fig. 5c, in 6-OHDA-treated cells, the increase of H3K27me3 induced by GSK-J4 was inhibited by GSK126 (*P* < 0.001, when comparing the 6-OHDA+GSK-J4 group with 6-OHDA+GSK-J4+GSK-126). However, MTT assay showed that there was no significant difference in the cell viabilities of these two groups (Fig. 5d). Next, we performed similar experiments to determine the role of H3K4me3. SH-SY5Y cells were treated with GSK-J4 (0.5 μM), followed by the treatment with 3-deazaneplanocin A (DZNep, 1 μM), which was reported to inhibit H3K4me3 levels^27^. As shown in Fig. 5e, H3K4me3 was inhibited in the presence of DZNep (*P* < 0.01, when comparing the 6-OHDA+GSK-J4 group with 6-OHDA+GSK-J4+DZNep). In addition, MTT assay showed that DZNep partially antagonized the neuroprotective effect of GSK-J4 (*P* < 0.05, when comparing the 6-OHDA+GSK-J4 group with 6-OHDA+GSK-J4+DZNep) (Fig. 5f). These data suggest that the increase of H3K4me3, but not that of H3K27me3, contributes to the neuroprotective effect of GSK-J4 against 6-OHDA treatment.

### H3K4me3 plays a role in iron metabolism under GSK-J4 treatment

**Fig. 6:**
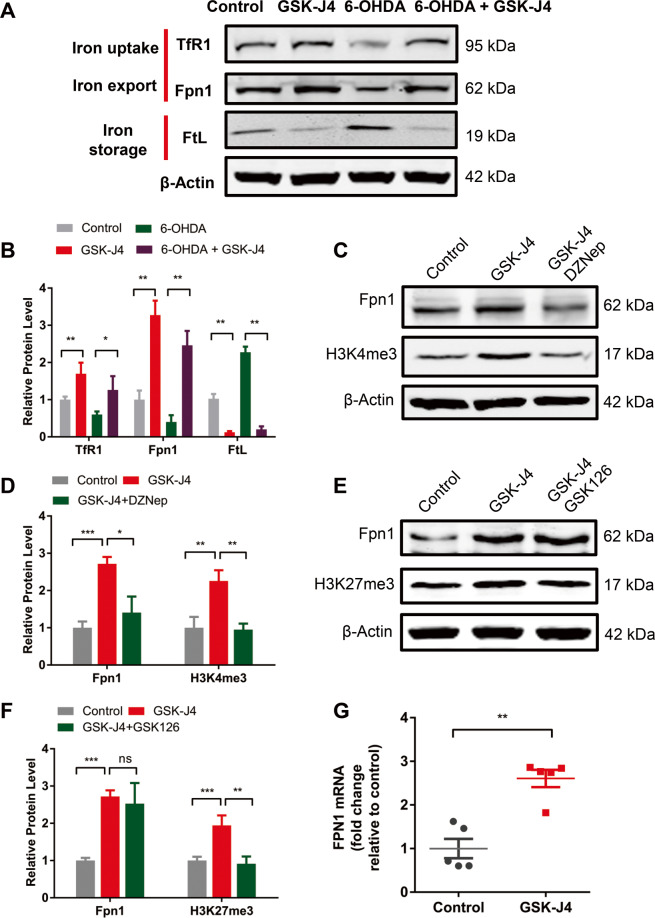
GSK-J4 rescues 6-OHDA-induced abnormal iron metabolism. **a**, **b** In the SH-SY5Y cell line, GSK-J4 treatment (0.5 μM, for 24 h) increased iron uptake proteins (TfR1) and the known iron export protein (Fpn1), and decreased the iron storage protein (FtL), not only in the basal condition, but also in the treatment of 6-OHDA. *P < 0.05; **P < 0.01. Data are presented as mean ± SEM. *n* = 3–4 for each group. **c**, **d** DZNep (1 μM) inhibited the GSK-J4-induced change of Fpn1 and H3K4me3, while **e**, **f** GSK126 (1 μM) failed to block the change of Fpn1 under GSK-J4 treatment (0.5 μM, for 24 h) although there was a decrease of H3K27me3. *P < 0.05; **P < 0.01; ***P < 0.001; ns, means no significant difference. Data are presented as mean ± SEM. *n* = 3–4 for each group. **g** RT-PCR showed GSK-J4 increased the mRNA level of Fpn1. *n* = 5 for each group. **P < 0.01. Data are presented as mean ± SEM.

Since GSK-J4 was found to selectively regulate iron metabolism in SH-SY5Y cells, while histone modifications is often locus type and cell type specific^28–30^. To fully elucidate the therapeutic mechanism of action of GSK-J4, we explored the relationship between H3K4me3 and iron homeostasis. First, in SH-SY5Y cells, Western blot analysis revealed that, when compared with the control cells, GSK-J4 treatment (0.5 μM, 24 h) significantly increased the levels of iron uptake proteins TfR1 (*P* < 0.01), the iron export protein Fpn1 (*P* < 0.01), and decreased the iron storage protein FtL (*P* < 0.01) (Fig. 6a, b). Together, these data are consistent with that increased iron export by Fpn1 is the primary cause of iron-suppressing effect of GSK-J4. Indeed, iron storage protein FtL was largely increased (*P* < 0.01) by 6-OHDA treatment when compared with control cells, while a marked decrease of FtL was found in the 6-OHDA plus GSK-J4 treatment group (*P* < 0.01, when compared with 6-OHDA-treated cells). Moreover, GSK-J4 could significantly prevent the 6-OHDA-induced decrease of Fpn1 and TfR1. In another set of experiments, it was found that the increase of Fpn1 could be inhibited by the addition of DZNep (decreasing H3K4me3) (Fig. 6c, d) while not by the addition of GSK126 (decreasing H3K27me3) (Fig. 6e, f). Consistent with the effect on iron metabolism, the effects of GSK-J4 on H3K4me3 and H3K27me3 were only found in SH-SY5Y cell line but not in HEK293 or HepG2 cell lines (Supplementary Fig. 2a, b). Furthermore, real-time PCR experiments showed that GSK-J4 could also upregulate the expression of Fpn1 at the mRNA level (Fig. 6g). These results together implicate that H3K4me3 plays a role in the iron metabolism under GSK-J4 treatment.

### Upregulation of H3K4me3 increases iron exporter Fpn1 and exhibits neuroprotection

**Fig. 7:**
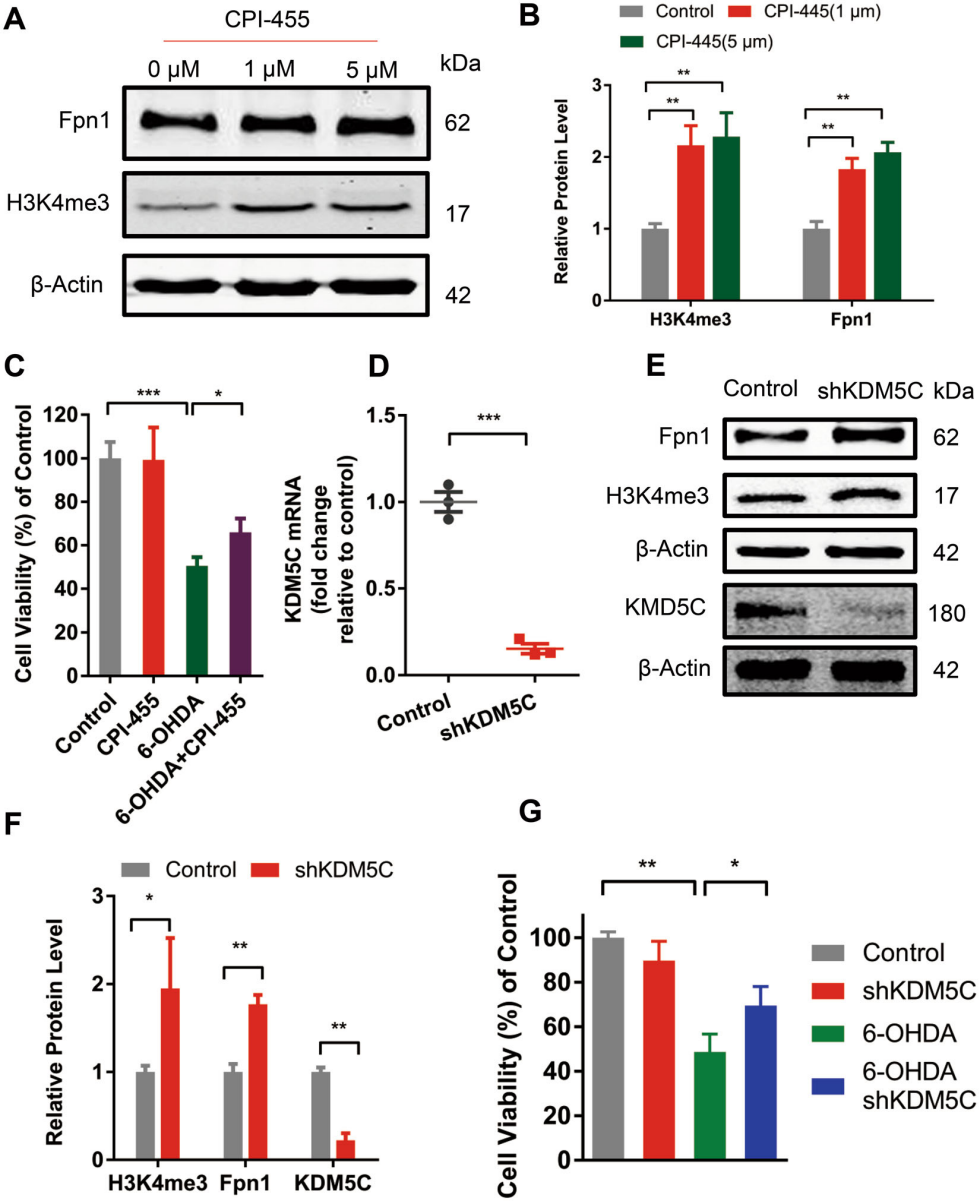
Upregulation of H3K4me3 increases iron exporter Fpn1 and exhibits neuroprotection. **a**, **b** CPI-455 treatment (24 h) increased the protein level of H3K4me3 and Fpn1 in SH-SY5Y cells. **c** MTT assay showed CPI-455 showed neuroprotection against the toxicity of 6-OHDA in SH-SY5Y cells. **d**–**g** SH-SY5Y cells were infected by AAV-KDM5C-silencing virus, and KDM5C was significantly decreased at the mRNA level as reflected by RT-PCR **d** and at the protein level by Western blot **e**, **f**. In addition, Fpn1 and H3K4me3 were increased in AAV-KDM5C-silencing virus infected cells **e**, **f**. Moreover, **g** silencing KDM5C showed a slight neuroprotection against 6-OHDA-induced PD model in SH-SY5Y cells. *P < 0.05; **P < 0.01; ***P < 0.001. Data are presented as mean ± SEM. *n* = 3–4 for each group in Western blot and *n* = 5 for each group in MTT assay.

To further probe the role of H3K4me3 in cell-specific iron regulation of GSK-J4, SH-SY5Y cells were pretreated with CPI-455, a specific inhibitor of KDM5, which could increase the global level of H3K4me3^31^. We found that CPI-455 (1 and 5 μM) caused a significant increase (over 2-fold) in the global level of H3K4me3 (*P* < 0.01, when compared with the control group, Fig. 7a, b). Under this condition, CPI-455 treatment also resulted in an increase in the iron exporter Fpn1. In addition, CPI-455 treatment improved cell viability in response to 6-OHDA exposure (Fig. 7c).

KDM5C is the main histone demethylase that can specifically alter H3K4 methylation in several cell types^31,32^. To further probe the role of H3K4me3 in regulating iron metabolism, the effect of AAV-KDM5C-silence virus was tested. As shown in Fig. 7d, KDM5C silencing strongly decreased KDM5C levels to 17% of control based on the mRNA level and to 16% of control based on the protein level (Fig. 7e, f).

Remarkably, significant increases of Fpn1 and H3K4me3 were observed under this condition (Fig. 7e, f). Also, KDM5C silencing improved the viability of cells under 6-OHDA treatment (Fig. 7g). Thus, KDM5C-mediated upregulation of H3K4me3 is involved in the increase of iron exporter (Fpn1) underlying the neuroprotective effect of GSK-J4.

## Discussion

In this study, we identified GSK-J4 as a potent and cell type-specific suppressor of intracellular labile iron, which confers neuroprotection from oxidative stress in 6-OHDA-induced PD model, alleviating Parkinsonian motor deficits. Importantly, we found that this effect is largely attributable to the upregulation of H3K4me3 by GSK-J4. A higher level of H3K4me3 expression leads to an increased iron export via the iron exporter Fpn1. Therefore, we discovered a novel and effective mechanism of neuron-specific suppression of iron level with clear clinical implication for neurodegenerative diseases.

The decrease in intracellular iron caused by GSK-J4 is likely due to alterations in iron transport and storage proteins. If the iron exporter (Fpn1) increases, it will cause a decrease in cellular iron and a corresponding decrease in iron storage (FtL) and an increase in iron uptake (TfR1). However, if the iron uptake TfR1 increases, it will cause an increase in cellular iron with a corresponding increase in FtL and an increase in Fpn1. Thus, it is reasonable to assert that an increase of Fpn1 is the initial effect of GSK-J4. In fact, our data showed that Fpn1 is epigenetically regulated in cells treated with GSK-J4. A major consequence of this change is the reduction in oxidative stress, which is believed to be a common mechanism involved in many neurodegenerative diseases, including PD^4,6,7,14^. In fact, clinical studies have found that elevated iron in SN can trigger and exacerbate oxidative stress, leading to DA neuronal death^7,14,16,17^. Consistently, in this study, GSK-J4 inhibited the increase of ROS, malondialdehyde (MDA), protein carbonyl, and lower levels of glutathione in 6-OHDA models. Also, GSK-J4 suppressed the direct oxidative inducer (H_2_O_2_) caused cell death in SH-SY5Y cells.

In this study, the iron-suppressive effect of GKS-J4 is cell type-specific and only trace amounts of this compound can significantly reduce the iron storage protein FtL. These findings strongly suggest that the effect of GSK-J4 cannot be solely explained by its ability to directly chelate iron. In fact, the most important discovery of the present study is the epigenetic cause of the iron-suppressive and neuroprotective effect of GSK-J4, as reflected by the involvement of H3K4me3. Cell-type specificity is known to be an important feature of epigenetic modification in regulating gene expression^30^. Our study indicated that the effects of GSK-J4 on H3K4me3 and H3K27me3 were only found in SH-SY5Y cell line but not in HEK293 or HepG2 cells. Although the reason for this cell specificity is not known, different microenvironments in different cell lines may underlie different responses to the treatment of GSK-J4. Further exploration on this issue is needed. It is noteworthy that, similar to the phenomenon we observed, Fpn1 could be regulated at both the transcription and post-translation level^33^, and in a cell-type-dependent manner at the transcription level^34^. GSK-J4 is currently regarded as a useful small molecular tool of epigenetic modification, especially for histone methylation, including the H3K4 and H3K27 trimethylation levels^19^. Methylation of histones can induce different changes in gene expression, depending on the specific site of methylation^35^. In general, trimethylation of H3K4 is associated with transcriptional activation of gene expression, while trimethylation of H3K27 is associated with transcriptional inhibition of gene expression^28,35^. With respect to regulation of iron level, GO gene analysis in KDM5 silencing in flies revealed its close relationship with the metal iron transport pathway^36^, which further indicated the potential role of H3K4me3 in the regulation of iron metabolism.

Most PD cases are idiopathic in nature and do not have a clear genetic link. On the other hand, since epigenetic modification could account for the interaction between gene expressions and environmental insults, epigenetic changes have recently been suggested to play a role in PD pathogenesis^37^. Among all examined epigenetic histone modifications in PD, histone methylation remains relatively unknown. In this study, it was found that 6-OHDA itself caused a significant decrease in H3K4me3 and H3K27me3. Indeed, other reports found that perinucleolar and perinuclear H3K27me3 levels decreased in the SN upon 6-OHDA injection^38^. Although previous studies have shown that L-DOPA can induce phosphorylation of H3K27meS28 in the striatum in the unilateral 6-OHDA-lesioned model, local changes in H3K27me3 and H3K4me3 have not been established^39^. This may be due to the different expression patterns between the different cells in these brain regions^40^. Our present findings strengthen the possibility that histone methylation may play a role in the pathogenesis of PD.

KDM5 is involved in regulating transcription and chromatin remodeling, mainly through the regulation of H3K4me3^32^. Among the KDM5 family, KDM5C is highly expressed in the central nervous system. Previous studies have shown that mutations in KDM5C are associated with X-linked mental retardation^41^. KDM5C-knockout mice showed abnormal dendritic arborization, spine anomalies, and altered transcriptomes. These mice also exhibited impaired social behavior, memory deficits, and aggression^42^. However, in some studies, silencing KDM5C, the most abundant form of KDM5 in brain, is associated with beneficial effects in neurodegenerative diseases such as Huntington’s disease, which increases the level of neurotrophic factor (BDNF) and synaptic proteins (SYP) through the increased level of H3K4me3 in Huntington’s disease^22^. In addition, it was found that the level of H3K4me3 in the striatum is significantly reduced in MPTP-induced mouse or nonhuman primate PD models^43^. These and our own findings are in notion with a significant role of KDM5–H3K4me3 in the pathogenesis and treatment of PD.

Chelating accumulated iron thus reducing oxidative stress has been considered a feasible treatment for PD^16,44^. Although several iron chelating agents such as DFO can effectively suppress neuronal loss in the SN and the striatum in several PD animal models^15,45^, due to their low efficiency in chelating iron, the existing iron chelating agents require a high dose when used, resulting in notable side effects. Besides, most of these current iron chelators cannot pass through the BBB, severely limiting their application in neurodegenerative diseases^17,44,46^. Compared with other iron chelators as a neuroprotective agent, GSK-J4 possesses several advantages. First, GSK-J4 exhibited a higher capacity to suppress free labile iron when compared with current iron chelators used in clinical practice, therefore minimizing side effects. Second, different from other iron chelating agents, GSK-J4 could suppress iron with cell-type specificity. Indeed, cell-specific iron chelators have not been reported as only organelle-specific chelating agents, such as mitochondrial iron chelators are available^47^. At present, GSK-J4 is the only agent that can specifically affect iron metabolism in neuronal cells. Finally, GSK-J4 has been reported to be able to pass through the BBB^48^.

In conclusion, we demonstrate a previously unappreciated mechanism of GSK-J4 in the selective iron suppression, conferring neuroprotection from oxidative stress in PD animal model. Our results not only facilitate the development of new drug targets that may eventually slow down, stop, or even reverse PD progression but also shed light on pathogenic mechanism of this disorder.

## Materials and methods

### Drugs and chemicals

The following chemical agents were used in the experiment: GSK-J4 (S7070, Selleck Chemicals), GSK-J1 (S7581, Selleck Chemicals), 6-OHDA (H116, SIGMA), H_2_O_2_ (H1009, SIGMA), CPI-455 (S8287, Selleck Chemicals), GSK126 (15415, Cayman Chemical), DZNep HCl (S7120, Selleck Chemicals).

### Animals

Sprague-Dawley (SD) rats weighing 320–350 g were used in this study. The animals were housed in standard cages with ad libitum access to both food and water. All animals were housed at a temperature of 25 °C under a 12-h light-on–light-off schedule. After that, all animals were randomly divided into different groups. All animal procedures were performed in accordance with the protocol approved by the Chinese University of Hong Kong Animal Ethics and Experimentation Committee.

### Cell culture

Cell cultures were prepared using the methods described previously^49^. In brief, undifferentiated SH-SY5Y cells and MES23.5 cells (kindly provided by Prof. Wei-dong Le, Shanghai Institutes for Biological Sciences, CAS) were grown in a 1:1 mixture of Dulbecco’s modified Eagle’s medium (DMEM) (10313021, Gibco, USA) and F-12 nutrient mixture (Ham12) (11765062, Gibco, USA) supplemented with 10% heat-inactivated fetal bovine serum (FBS) (10082147, Gibco, USA). In addition, the human hepatocellular carcinoma (HepG2) cell line and HEK293 cells (human embryo kidney cells), purchased from the American ATCC Cell Line Center, were maintained as an adherent cell line in DMEM supplemented with 10% FBS, 2 mmol/l l-glutamine (Gibco, USA), and 1× nonessential amino acids (Gibco, USA). Cells were passaged as needed using 0.5% trypsin–EDTA (Gibco, USA). All culture mediums contained 100 units/ml of penicillin (Gibco, USA) and 100 mg/ml of streptomycin (Gibco, USA). All cells were cultured at 37 °C in a 5% CO_2_ and 95% air-humidified atmosphere.

### MTT assay

To study the effect of GSK-J4 on neuroprotection, MTT assays were performed to assess cell viability as previously described^49^. In brief, SH-SY5Y cells were cultured in the 96-well plates at a density of 1 × 10^5^ cells per well at 37 °C. Two days later, cells were placed in a fresh medium with different treatments. After a particular treatment, cell viability was assessed by the MTT assay (11465007001 ROCHE, Cell Proliferation Kit I (MTT)). First, the supernatant in each culture well was removed and the wells were washed with PBS twice. 20 μl MTT solution (5 mg/ml in PBS) was added to each well (containing 100 μl culture medium). After incubating for 4 h, the medium was removed carefully. Then, 150 μl DMSO was added to each well and shaken for 10 min to allow the crystals to be fully melted. Finally, the absorbance intensity was measured at 490 nm with a microplate reader (Bio-RAD 680, USA) and together with a reference wavelength of 620 nm. Control wells (cells with the same concentration of drug in medium) were set at the same time. The cell viability (%) was expressed as a percentage relative to the control group.

### PD animal models

#### 6-OHDA-induced PD rat model

6-OHDA PD rat model was established according to our method described previously^50,51^. Briefly, SD rats were deeply anesthetized with ketamine (75 mg/kg, i.p.) and xylazine (6 mg/kg, i.p). They then received stereotaxic injections of 6-OHDA (2 μl of 4 μg/μl) dissolved in 0.9% saline containing 0.2 mg/ml ascorbic acid to the unilateral MFB, at a site relative to the bregma: anteroposterior (AP), −4.4 mm; mediolateral (ML), +1.2 mm; and dorsoventral (DV), 8.0 mm from dura. The injection was administered within 5 min and the Hamilton syringe needle was kept in situ for 5 min. The animals were monitored for 2 h post-surgery before returning to the animal housing facility. Separate groups of control rats received saline sham injections following the same procedures.

### Behavioral test

#### Cylinder test

To evaluate motor dysfunction, a forelimb use asymmetry test (the cylinder test) was used^52^. In short, 14 days after injection of 6-OHDA, animals were placed in a quiet, well-lit room at least half an hour in advance. Then, the rats were placed in a clean, open-top plexiglass cylinder (diameter: 20 cm, height: 30 cm). The behavior was recorded for 5 min for each session. Three observers who were blind to the treatment scored the numbers that the animal used the left and the right forelimbs to touch the cylindrical walls.

#### Apomorphine-induced circling test

Apomorphine-induced circling test was used to reflect DA denervation in rodents with unilateral nigrostriatal lesion^50,52^. In brief, 14 days after surgery, rats were injected intraperitoneally with apomorphine (M8750 Sigma, 5 mg/kg) and placed in a clean open arena (120 × 120 cm). The whole experiment was performed in a quiet, well-lit room. Thirty minutes after the injection, the rats were recorded for 10 min. The number of rotations towards the contralateral side of lesion was counted.

### Immunostaining

Immunostaining was carried out as reported before with some modifications^53^. Briefly, the rats having received different treatments were deeply anesthetized with 4% chloral hydrate (0.4 mg/g, i.p.) followed by perfusion with normal saline through the left ventricle. The brains were removed and post-fixed in 4% paraformaldehyde (PFA) followed by cryoprotection with serial sucrose solutions. After that, brain tissues were immersed in cryo-embedding media (OCT) and brain sections (30 μm) that contained the SN (AP: −4.80 mm to AP: −6.12 mm from bregma) were prepared by a cryotome. Every fourth section was stained for TH and analyzed. The brain slices were then incubated in blocking solution followed by overnight incubation at 4 °C with primary antibodies such as anti-TH (1:1000, Millipore). After washing with PBS, the slides were incubated with Alex 546 or FITC-conjugated secondary antibody for 1 h at room temperature. The nuclei were counterstained with DAPI. The slides with immunofluorescence staining were visualized with a Nikon C-1 confocal laser scanning microscope (Nikon). The number of TH-positive cells as well as the areas of SN (in mm^2^) were analyzed by using ImageJ software (from National Institutes of Health and available at http://rsb.info.nih.gov/ij/).

### Western blotting

Brain tissues or cells were washed and homogenized in RIPA lysis buffer and then sonicated. Aliquots of the extract containing about 30 μg of protein were loaded and ran on a single track of 10–12% SDS–PAGE under reducing conditions and subsequently transferred to a pure nitrocellulose membrane (Bio-Rad). The blots were blocked and incubated with primary antibodies anti-H3K4me3 (#9751, Cell Signaling Technology, 1:1000), anti-H3K27me3 (07-449, Merck Millipore, 1:1000), anti-KDM5C/SMCX/Jarid1C (ab190180, ABCAM 1:1000), anti-Ferritin-L (ab69090, ABCAM, 1:1000), anti-transferrin receptor 1 (13-6800, Thermo Fisher Scientific, 1:1000), anti-ferroportin/SLC40A1 antibody (NBP1-21502, Novus Biologicals, 1:1000), anti-Bcl2 (ab692, ABCAM, 1:1000), and anti-cleaved caspase-3 (#9664, Cell Signaling Technology, 1:1000) overnight at 4 °C. After the incubation, the blots were washed and then incubated with goat anti-rabbit or anti-mouse IRDye 800 CW secondary antibodies (1:5000, Li-Cor) for 1 h at room temperature. The intensities of specific bands were detected and analyzed by Odyssey infrared imaging system (Li-Cor). To ensure even loading of the samples, the same membrane is probed with rabbit anti-β-actin polyclonal antibody at a 1:5000 dilution.

### Real-time PCR

Total RNA was isolated from cultured cells using Trizol^®^ Reagent (Invitrogen Corp., Carlsbad, CA) following the manufacturer’s protocol. RNA concentrations were evaluated utilizing spectrophotometer and the purity of the RNA was assessed by calculating the ratio of A260–A280 nm signals. RNA was reversely transcribed using Superscript II Reverse Transcriptase (Invitrogen Corp., Carlsbad, CA) and Oligo(dT) Primer (Invitrogen Corp., Carlsbad, CA) following the manufacturer’s instruction. The cDNA for primer design for RT-PCR was previously reported. Primer sequences were as follows:

KDM5C forward primer, 5′-GAGGTGACCCTGGATGAGAA-3′, and KDM5C reverse primer, 5′-CAGGAGCTGAGGTCTGAAC-3′;

FPN1 forward primer, 5′-CCA CCT GTG CCT CCC AGA T, and FPN1 reverse primer, 5′-CCC ATG CCA GCC AAA AAT AC-3′.

cDNA amplification and detection were fulfilled using MyiQ Real-time PCR Detection System (Bio-Rad Laboratories, CA) and iQ SYBR Green Supermix (Bio-Rad Laboratories, CA). The initial denaturation was 95 °C for 5 min, then 95 °C for 10 s followed by 60 °C for 45 s, which cycled for 40 times.

The β-actin cDNA (3′-primer, 5′-CTCTCAGCTGTGGTGGTGAA-3′; 5′-primer, 5′-GTCGTACCACTGGCATTGTG-3′) was simultaneously amplified as the internal control. Relative quantification exploited the comparative Cx method. The mRNA level of KDM5C of each sample was normalized to that of the level of β-actin and control group samples were used as calibrator. Relative mRNA level was calculated by the 2^−ΔΔCT^ method.

### DAB-enhanced Perls’ iron staining

DAB-enhanced Perls’ iron staining was performed as described previously^54^. Briefly, the slides were fixed with 4% PFA for 10 min. After three washes with PBS, the slides were incubated in Perls’ solution containing 8% potassium ferrocyanide and an equal volume of 1.2 mmol/l hydrochloride acid solution for 16 h at 4 °C and then washed with deionized water three times, followed by dehydration through 95% alcohol and mounting with xylene. Afterwards, the sections were washed and incubated in a solution of DAB to enhance the signals. Iron staining was observed under a Nikon Eclipse TE2000-U microscope (Nikon, UK).

### Calcein-AM assay

The ferrous iron measurement on cell was performed using calcein-AM method as previously described, with some modifications^55^. Briefly, SH-SY5Y cells were grown in DMEM supplemented with 10% heat-inactivated fetal bovine serum, and cultured at 37 °C under humidified 5% CO_2_ atmosphere. Cells were treated with ferric ammonium citrate (FAC) or GSK-J4 or GSK-J4 plus FAC for 12 h in serum-free media. Then they were washed with PBS twice, followed by the addition of calcein-AM (50 nM), and incubated for 30 min at 37 °C. After that, fluorescence was measured using a microplate fluorometer (Thermo Fisher Scientific Inc).

### ROS, protein carbonylation determination

ROS were measured by the cell-permeant 2′,7′-dichlorodihydrofluorescein diacetate (H_2_DCFDA) assay as previously described with minor modifications^56^. Briefly, brain, cell, or protein extraction were incubated with 5 μM H_2_DCFDA in dark at 37 °C for 30 min and then washed three times with PBS. DCF fluorescence was observed under Nikon C-1 confocal laser scanning microscope and quantified using a fluorescence microplate reader (LS55 fluorescence Spectrometer, PerkinElmer) with an excitation/emission wavelength of 485/515 nm in 96-well fluorescent plates. Protein carbonyls were measured by using an OxyBlotTM protein oxidation detection kit (Chemicon), in which carbonyl groups in the protein side chains are derivatized to 2,4-dinitrophenylhydrazone (DNP) by reaction with 2,4-dinitrophenylhydrazine (DNPH).

### MDA level

Lipid peroxidation was determined in total brain lysates using the N-methyl-2-phenylindole based LPO-586™ lipid peroxidation kit (Oxis International, OR, USA)^57^. This assay measures the level of MDA. Standard curves of MDA were established using 1,1,3,3 tetramethocypropane. To minimize non-specific oxidation during sample preparation, 5 mm butylated hydroxytoluene (BHT dissolved in acetonitrile) was added to the extraction buffer. The assay was performed in triplicates according to manufacturer’s recommendation using 4 mg of total brain lysates per reaction, and the results were calculated as picomoles of MDA per mg of protein. Briefly, 200 µl of brain lysates (4 mg) was added to 10 µl of 0.5 M BHT and 650 µl of 0.1 mM N-methyl-2-phenylindole, followed by gentle mixing. For MDA measurement, 150 µl of reagent grade HCl (∼36%) was added. The sample was mixed and incubated for 1 h at 45 °C. After incubation, the sample was centrifuged, and the supernatant was extracted and measured at 586 nm.

### GSH level

Total reduced GSH was measured as described previously^57^. Briefly, the acid-soluble fraction was obtained from cell lysates and tissue homogenates by adding perchloric acid to a final concentration of 3%, followed by centrifugation at 14,000 × *g* for 10 min. The acid-soluble fraction was neutralized to pH 7 with 0.5 M KOH/50 mM Tris. After the removal of precipitate (potassium perchlorate) by a second centrifugation, 50 μl aliquots of sample was combined with 100 μl of reaction mixture consisting of 2.5 ml of 1 mM 5,5′-dithio-bis-(2-nitrobenzoic acid) (DTNB), 2.5 ml of 5 mM NADPH, 2.5 ml of phosphate buffer solution (100 mM NaPO4, pH 7.5, 1 mM EDTA), and glutathione reductase (5 U/ml final volume). GSH-mediated reduction of DTNB was measured at 412 nm at 30 s intervals over 30 min. GSH content was normalized to protein.

### Construction of AAV virus

The coding silence sequence of KDM5C will be first ligated into a shuttle plasmid pAAV with EGFP-tag (Addgene). Insertion of the coding sequence was then verified by sequencing. The positive recombinant plasmid was co-transfected with pAAV-RC, pAAV-helper (Addgene) into HEK293 cells. AAV was harvested 3 days later and designated as “AAV-KDM5C”. The titers of the virus were measured according to standard procedure.

### Statistical analysis

All data were presented as mean ± standard error of the mean (SEM). Graphpad Prism was used for statistical analysis. Unpaired Student’s *t* test or one-way ANOVA with a post hoc Tukey’s test was performed, as appropriate, to determine significant differences between groups. A probability value of *P* < 0.05 was taken to be statistically significant. All the experiments consisted of at least three replicates.

## Supplementary information

Supplementary Figure 1

Supplementary Figure 2

Supplementary Information

## Data Availability

The data that support the findings of this study are available from the corresponding author (Y.K.) upon reasonable request.
