# Supplementary figures and images for: Therapeutic effect of a histone demethylase inhibitor in Parkinson’s disease
Source: Cell Death Dis. 2020 Oct 28;11(10):927. doi: 10.1038/s41419-020-03105-5 (PMC7595123; doi:10.1038/s41419-020-03105-5)

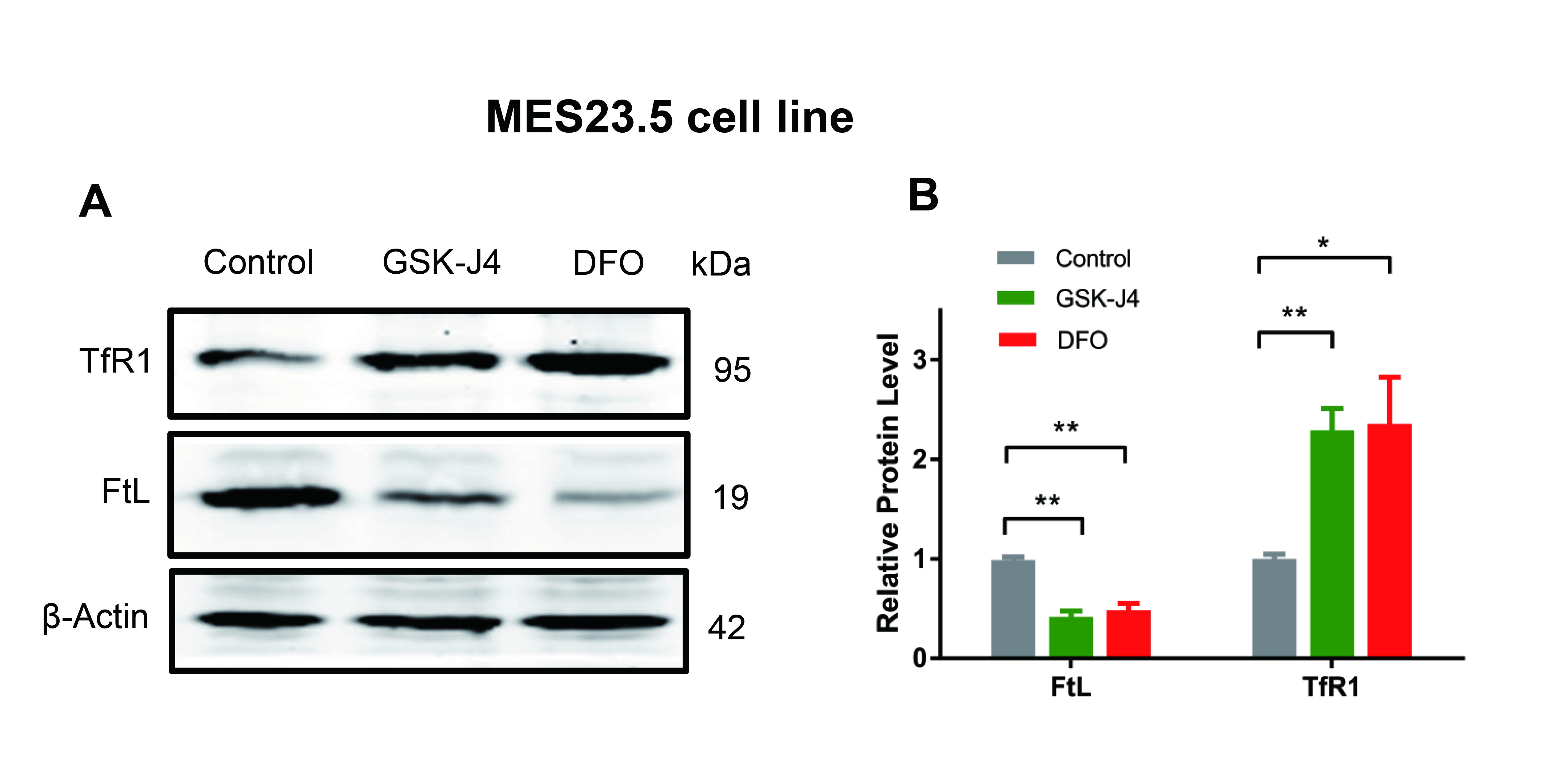

Supplement: Supplementary file 1 — Supplementary Figure 1 [file 41419_2020_3105_MOESM1_ESM.tif]

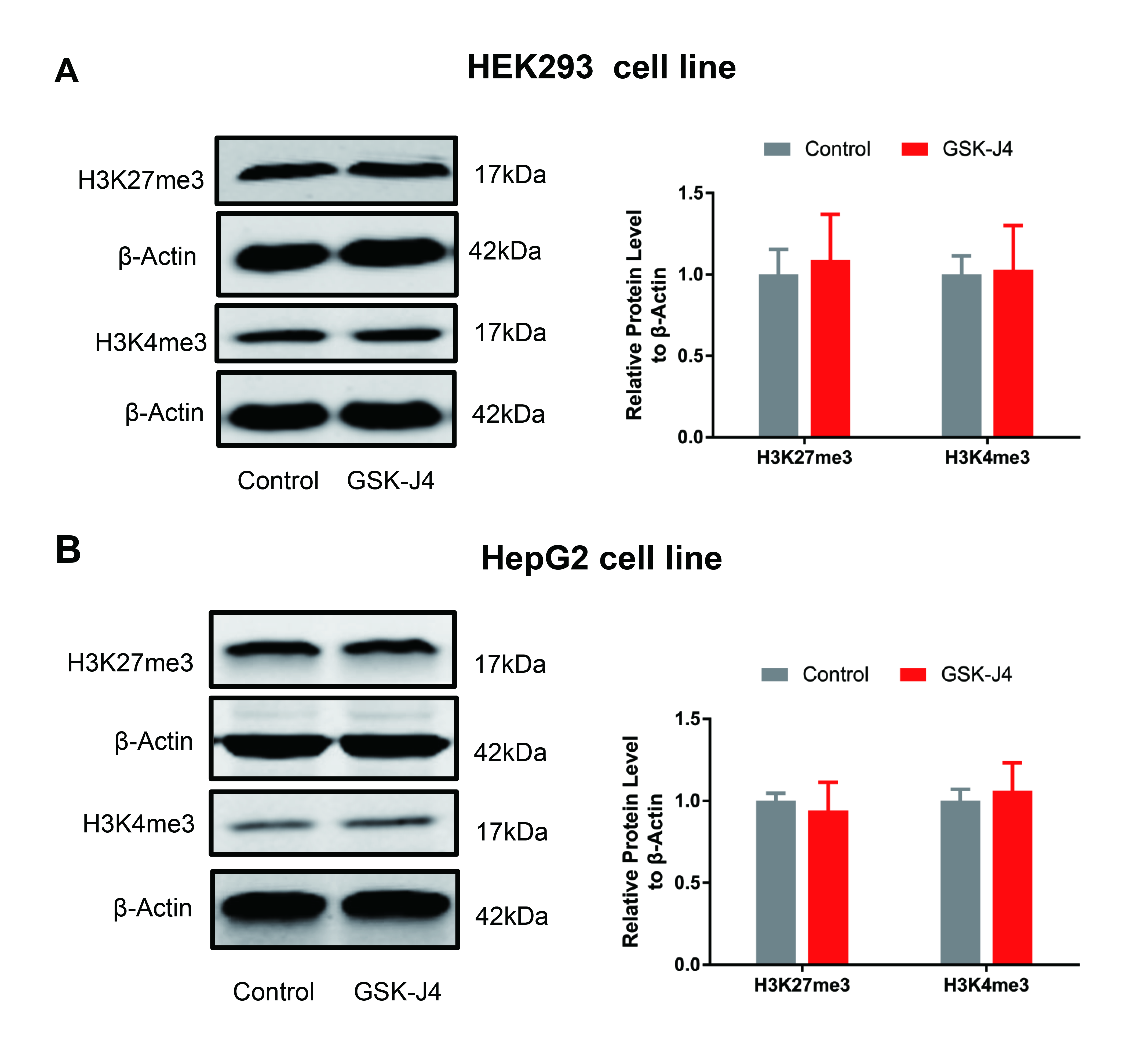

Supplement: Supplementary file 2 — Supplementary Figure 2 [file 41419_2020_3105_MOESM2_ESM.tif]
