# Supplementary material for: Therapeutic effect of a histone demethylase inhibitor in Parkinson’s disease
Source: Cell Death Dis. 2020 Oct 28;11(10):927. doi: 10.1038/s41419-020-03105-5 (PMC7595123; doi:10.1038/s41419-020-03105-5)
Supplement: Supplementary file 3 — Supplementary Information [file 41419_2020_3105_MOESM3_ESM.docx]

**Supplementary Figure 1. Effects of GSK-J4 on FtL and TfR1 in MES23.5 dopaminergic cell line.**

(a-b) GSK-J4 treatment (0.5 μM, 24 hours), like the common iron chelator DFO (50 μM, 24 hours), caused a decrease of FtL and an increase of TfR1 in the MES23.5 cell line, as revealed by Western blotting. The relative protein levels of FtL and TfR1 were normalized to beta-actin. *, *P*<0.05; **, *P*<0.01. Data are presented as mean±SEM. n=3 for each group.

**Supplementary Figure 2. No significant changes in histone methylation with treatment of GSK-J4 on HEK293 cells and HepG2 cells.**

Western blot showed that no significant changes in the levels of H3K4me3 and H3K27me3 under GSK-J4 treatment in the (a) HEK293 cell line and (b) HepG2 cell line. Data are presented as mean±SEM. n=3 for each group.
